# Brain Structural Correlates of Reward Sensitivity and Impulsivity in Adolescents with Normal and Excess Weight

**DOI:** 10.1371/journal.pone.0049185

**Published:** 2012-11-21

**Authors:** Laura Moreno-López, Carles Soriano-Mas, Elena Delgado-Rico, Jacqueline S. Rio-Valle, Antonio Verdejo-García

**Affiliations:** 1 Department of Personality, Evaluation and Psychological Treatment, University of Granada, Granada, Spain; 2 Department of Psychiatry, Bellvitge University Hospital-IDIBELL, Barcelona, Spain; 3 Carlos III Health Institute, Centro de Investigación Biomédica en Red de Salud Mental (CIBERSAM), Granada, Spain; 4 Department of Nursing, University of Granada, Granada, Spain; 5 Institute of Neurosciences Federico Olóriz, University of Granada, Granada, Spain; University of Missouri-Kansas City, United States of America

## Abstract

**Introduction:**

Neuroscience evidence suggests that adolescent obesity is linked to brain dysfunctions associated with enhanced reward and somatosensory processing and reduced impulse control during food processing. Comparatively less is known about the role of more stable brain structural measures and their link to personality traits and neuropsychological factors on the presentation of adolescent obesity. Here we aimed to investigate regional brain anatomy in adolescents with excess weight vs. lean controls. We also aimed to contrast the associations between brain structure and personality and cognitive measures in both groups.

**Methods:**

Fifty-two adolescents (16 with normal weight and 36 with excess weight) were scanned using magnetic resonance imaging and completed the Sensitivity to Punishment and Sensitivity to Reward Questionnaire (SPSRQ), the UPPS-P scale, and the Stroop task. Voxel-based morphometry (VBM) was used to assess possible between-group differences in regional gray matter (GM) and to measure the putative differences in the way reward and punishment sensitivity, impulsivity and inhibitory control relate to regional GM volumes, which were analyzed using both region of interest (ROI) and whole brain analyses. The ROIs included areas involved in reward/somatosensory processing (striatum, somatosensory cortices) and motivation/impulse control (hippocampus, prefrontal cortex).

**Results:**

Excess weight adolescents showed increased GM volume in the right hippocampus. Voxel-wise volumes of the second somatosensory cortex (SII) were correlated with reward sensitivity and positive urgency in lean controls, but this association was missed in excess weight adolescents. Moreover, Stroop performance correlated with dorsolateral prefrontal cortex volumes in controls but not in excess weight adolescents.

**Conclusion:**

Adolescents with excess weight have structural abnormalities in brain regions associated with somatosensory processing and motivation.

## Introduction

Overweight and obesity are the ultimate consequence of an energy imbalance between consumed and expended calories. Nevertheless, the fact that -in the context of an unlimited access to food- not everyone becomes obese indicates that there are important individual differences in the susceptibility to develop such disorders. Although a number of psychological factors have been proposed to explain the development and maintenance of obesity [1], in the past few years, the motivational traits associated with reward and punishment sensitivity, and the personality and neuropsychological dimensions associated with impulse control, have been highlighted as relevant modulators of such susceptibility [2], [3]. The impact of these factors on eating behaviour seems to be particularly influential during adolescence [4], [5], a developmental period in which both motivational tendencies and impulse control skills strongly modulate goal-directed behaviour [6].

The study of the brain structures associated with these motivational, personality and neuropsychological variables in obese adolescents could provide more sensitive information about excess weight during adolescence, since regional brain anatomy indices may be considered a more stable measurement ultimately linked to both personality and cognitive modulators associated to the development of particular disorders [7]. Previous evidence from structural imaging studies have revealed that obese adolescents have lower total gray matter (GM) volumes and reduced regional GM volumes in the orbitofrontal cortex compared to lean controls [8], [9]. Moreover, in the Yokun et al. [8] study, higher body mass indices (BMIs) were correlated to volume changes in brain regions involved in reward processing (striatum), memory (middle temporal/parahippocampal gyri), and somatosensory processing (rolandic operculum), whereas reduced regional GM volumes in the prefrontal cortex correlated with steeper rates of BMI increase at 1-year follow-up. Furthermore, Maayan et al. [9] found that obese adolescents were characterized by increased trait disinhibition scores and poorer cognitive control, and that both features correlated with the reduced GM volumes in the orbitofrontal cortex. These findings indicate that volumetric brain measures are useful to characterize the neurobiological underpinnings of adolescent obesity, and that brain structural volumes are associated with both disease-specific features (e.g., BMI) and impulsive personality and cognitive control functions.

Such findings are broadly in agreement with the results from functional imaging studies in obese adolescents and adults, in which these regions seem to play different roles. For example, during the processing of food rewards striatal activation is decreased whereas activations of prefrontal and somatosensory regions are increased in obese adolescents [10], [11]. There is also evidence of increased resting activity in the somatosensory cortices of obese adults [12]. Moreover, the hippocampus is selectively engaged during gastric stimulation and this activation correlates with emotional eating and lack of control in obese adults [13]. Such results have led to hypothesize that decreased striatal functioning and increased somatosensory functioning may be associated with increased reward sensitivity in obese individuals, whereas increased hippocampal and prefrontal reactivity may relate to the balance between the emotional appeal of food and the cognitive control of eating behaviour [10], [12], [13].

In this study we used magnetic resonance imaging (MRI) and voxel based morphometry (VBM) procedures to assess regional brain anatomy in adolescents with excess weight. The aim of the study was twofold: firstly, to detect regional GM volume differences between adolescents with excess weight and adolescents with normal weight, and secondly, to examine possible differences in the way reward and punishment sensitivity, impulsive personality and cognitive control relate to regional GM volumes in both groups. We performed both a region of interest (ROI) and a whole-brain analyses approach. The ROIs were selected based on previous evidence of their involvement in adolescent obesity, and included the prefrontal cortex, the somatosensory cortices, the medial temporal lobe (including hippocampus), and the striatum. In agreement with previous evidence, we hypothesized that adolescents with excess weight will have decreased regional GM in the prefrontal cortex, whereas regional volumes of the striatum and the somatosensory regions will be related to reward sensitivity, and regional volumes of the prefrontal cortex will correlate with impulsivity and cognitive control.

## Methods

### 1. Participants

Fifty-two adolescents (12–17 years old) participated in the study. The participants were initially classified as adolescents with normal weight (n = 16, mean BMI = 20.26, SD = 2.8), overweight (n = 16, mean BMI = 24.85, SD = 1.42) or obesity (n = 20, mean BMI = 31.46, SD = 2.91) according to their BMI following the International Obesity Task Force (IOTF) criteria defined by Cole et al. [14]. However, since we did not find significant differences between the excess weight groups (overweight vs. obesity) in any of the psychological or imaging variables assessed, we decided to merge these two groups in a single “excess weight group”. Participants were recruited through educational centers and the endocrinology service of the hospital “Virgen de las Nieves” in Granada (Spain). Selection criteria were: (i) age between 12–17 years old, (ii) absence of a positive eating disorder history (Eating Disorder Inventory, EDI-2) [15], (iii) absence of personality disorders assessed by the Millon Adolescent Clinical Inventory (MACI) [16], and (iv) absence of past history or current existence of relevant medical problems (based on clinical history and a blood test). For both groups, evidence of significant abnormalities on MR images, contraindications to MRI scanning (including claustrophobia and implanted ferromagnetic objects) and history of loss of consciousness (LOC) for longer than 30 minutes or LOC with any neurological consequence were also exclusionary.

This study was approved by the Ethics Committee of the University of Granada. All subjects and their parents provided written informed consent before participating in the study.

### 2. Instruments and assessment procedures

Assessments were conducted across two independent sessions. During the first session we administered the personality and cognitive measures (see descriptions below), together with a battery of cognitive tests whose results will be reported separately. The second session involved the MRI scanning, which lasted approximately 15 minutes.

#### 2.1 Measure of reward and punishment sensitivity

*Sensitivity to Punishment and Sensitivity to Reward Questionnaire (SPSRQ):* This questionnaire is a self-report measure made up of 48 items, half of which assess the participant's appetitive motivational system, or reward sensitivity, and the other half the avoidance motivational system, or punishment sensitivity [17]. The reward and punishment sensitivity scales are reported to show adequate internal consistency, as well as convergent, construct and discriminate validity [18].

#### 2.2 Measure of impulsivity

*UPPS-P Scale* [19], [20]: This is a 59-item inventory designed to measure five distinct personality pathways to impulsive behavior: sensation seeking, (lack of) perseverance, (lack of) premeditation, negative urgency and positive urgency. The first 4 dimensions were included in the original version of the UPPS-P scale [19]; the fifth dimension has been included on the basis of recent work by Cyders et al. [21], and Smith et al. [22]. Each item on the UPPS-P is rated on a 4-point scale ranging from 1 (*strongly agree*) to 4 (*strongly disagree*). Sensation seeking (12 items) incorporates two aspects: 1) a tendency to enjoy and pursue activities that are exciting, and 2) an openness to trying new experiences that may or may not be dangerous; (lack of) perseverance (10 items) refers to an individual's ability to remain focused on a task that may be boring or difficult; (lack of) premeditation (11 items) refers to the tendency to think and reflect on the consequences of an act before engaging in it; and finally urgency refers to the tendency to experience strong impulses under conditions of negative affect (negative urgency –12 items) or positive affect (positive urgency –14 items). We obtained the total scores of each of these five UPPS–P dimensions for analyses. The Spanish version of the UPPS–P Impulsive Behavior scale have showed adequate levels of reliability and validity and is considered an useful instrument for assessment of impulsivity in Spanish-speaking population [20].

#### 2.3 Measure of inhibitory control

*Color-Word Interference Test–Stroop* (Delis–Kaplan Executive Functions System) [23]: The test consists of three different parts, each containing 50 items. Part 1 (Colour Naming) presents patches of colors and participants have to name them as quickly and accurately as possible. Part 2 (Reading) presents the words “red”, “blue” and “green” printed in black ink, and participants have to read the words aloud. Part 3 (Inhibition) introduces the interference effect: the words “red”, “blue” and “green” are printed in incongruent colors, and participants have to name the color and ignore the word. The main dependent variable derived from this test was Inhibition (time to complete Part 3 – time to complete Part 1). This test has showed adequate levels of reliability and validity and have been widely used in neuropsychology practice as a measure of inhibition and switching skills [23], [24].

### 3. MRI acquisition and pre-processing

Participants were scanned on a 3T whole body MRI scanner (Phillips Achieva X-series) operating with an eight-cannel phased array head coil. For each participant, a 3D volume was acquired using a T1-weighted turbo-gradient-echo sequence (3D-TFE) in the sagittal plane, with a 0.94×0.94×1.0 mm resolution (160 slices, FOV = 240×240 mm^2^, matrix 256×256), TR = 8.3 ms, TE = 3.8 ms, TI = 1022.6264 ms, and flip angle = 8°. This sequence was optimal for reducing motion sensitivity, susceptibility artifacts and field inhomogeneities.

Structural imaging data were pre-processed and analyzed using statistical parametric mapping 8 (SPM8) (http://www.fil.ion.ucl.ac.uk/spm) implemented in Matlab R2007b (MathWorks, Natick, MA, USA). We used the VBM8 toolbox (http://dbm.neuro.uni-jena.de/vbm/) to segment raw images and extract probabilistic maps of GM; normalize GM segments (using DARTEL normalization) to a GM template in MNI space; modulate normalized GM images with the Jacobian determinants (derived from the flow-fields of the normalization step) to restore volumetric information; and finally smooth images with a 3-D Gaussian filter of 8 mm full width at half maximum (FWHM).

## Data Analysis

### 1. Measures of reward/punishment, impulsivity and inhibitory control

We first analyzed the assumption of normal distribution of dependent variables using Kolgomorov-Smirnov tests. Likewise, we also assessed the homogeneity of variances between the study groups by means of Levene's tests. Both assumptions were met and therefore we conducted independent-sample t-tests to examine between-group differences in reward/punishment sensitivity, impulsivity and inhibitory control using SPSS 15.0 for Windows (SPSS Inc., Chicago IL). Significance threshold was set at p<0.05.

### 2. Image analysis

#### 2.1 GM differences between normal weight and excess weight groups

The general linear model was used to conduct between-group voxel-wise comparisons within SPM8. Group differences in regional GM volumes were tested using both a ROI and a whole-brain approach. Regarding ROI analyses, the ROIs selected were the orbitofrontal cortex, the dorsolateral prefrontal cortex, the somatosensory cortices (including SI and SII), the medial temporal lobe, and the striatum (all regions were assessed bilaterally). We used the WFU Pickatlas [25] to delineate these regions and create image masks that were used to restrict voxel-wise analyses to the region of interest (thus applying Small Volume Correction (SVC) procedures). In these analyses, the total volume of GM (TVGM) was modeled as a linear confound to account for global volume variability, and although study groups did not significantly differ in gender, to fully discard a potential impact of the apparent gender imbalance between our study groups, we also included this variable as a confounding covariate. Regarding whole brain analyses, we used the same statistical model, although the analyses were not restricted to any particular region. Significance threshold was set at p<0.05 after family-wise error (FWE) correction for multiple comparisons across the region of interest (pFWE-SVC<0.05) or across the whole brain (pFWE<0.05).

#### 2.2 Correlation analyses with personality and neuropsychological scores

Correlations between regional GM volumes and the scores of the different scales were also assessed within SPM8 by means of independent sample t-tests, in where the score of interest was modeled in interaction with the variable group (excess weight vs. normal weight participants). Confound variables and the significance thresholds were the same as above. Likewise, we also applied a ROI approach followed by a whole-brain analysis. Correlations were voxel-wise assessed within each group, and regions where significant findings were detected were further investigated to ascertain the existence of a between-group interaction in the pattern of correlations; that is, to verify that correlations were uniquely present in one of the study groups.

## Results

### 1. Sample characteristics

The participants' demographic characteristics – classified as normal weight vs. excess weight – are summarized in Table 1. The excess weight and normal weight groups were statistically matched on gender, age, years of education and socioeconomic status. As expected, relative to normal weight participants, excess weight participants had significantly greater weight (t_50_ = −5.385, p<0.005) and BMI (t_50_ = −7.371, p<0.005).

**Table 1:** Sociodemographic and biometric characteristics of study subjects.

	Normal weight (n = 16)	Excess weight (n = 36)^a^	Test
Age (years)	14.13 (1.36)	14.22 (1.4)	(t_50_ = −0.162, 0.872)
Years of education	10.13 (1.36)	10.19 (1.45)	(t_50_ = −0.162, 0.872)
Gender (male/female)	7/9	10/26	(*x* ^2^ = 1.284, 0.257)
SES (annual income €)^b^			(*x* ^2^ = 6.400, 0.171)
0–11.533 €	3	2	
11.533–18.200 €	2	11	
18.200–26.548 €	5	17	
26.548–41.294 €	3	2	
41.294–5.585.000 €	2	3	
Height	161.82 (9.87)	161.82 (7.55)	(t_50_ = 0.001, 0.999)
Weight	53.33 (11.02)	75.19 (14.45)	(t_50_ = −5.385, 0.000)
BMI^c^	20.26 (2.8)	28.53 (4.07)	(t_50_ = −7.371, 0.000)

a The excess weight group is composed of participants originally classified as having overweight (n = 16) or obesity (n = 20) according to the International Obesity Task Force criteria;

b SES: Socioeconomic status. Quintiles for SES are defined according to data from the Financial Survey for Spanish Families, http://www.bde.es/webbde/es/estadis/eff/eff.html;

c BMI: Body mass index.

### 2. Reward/punishment sensitivity, impulsivity and inhibitory control measures

There were no significant between-group differences in any of the measurements assessed (Table 2).

**Table 2:** Between-group comparison of impulsivity and SPSRQ scores.

	Normal weight (n = 16)	Excess weight (n = 36)	Test
SPSRQ^a^			
*Reward sensitivity*	11.25 (5.42)	9.28 (4.27)	(t_50_ = 1.414, 1.972)
*Punishment sensitivity*	11.06 (4.77)	9.47 (5.02)	(t_50_ = 1.071, 1.59)
UPPS-P			
*Sensation seeking*	32.94 (6.5)	28.94 (7.19)	(t_50_ = 1.414, 1.972)
*Lack of perseverance*	23.69 (5.3)	21.75 (4.34)	(t_50_ = 1.071, 1.59)
*Lack of premeditation*	26.63 (5.35)	25.58 (5.91)	(t_50_ = 1.414, 1.972)
*Negative urgency*	26.38 (7.59)	26.53 (7.1)	(t_50_ = 1.071, 1.59)
*Positive urgency*	24.25 (7.02)	24.36 (8.11)	(t_50_ = 1.414, 1.972)
Stroop			
*Inhibition*	12.25 (2.35)	11.75 (2)	(t_50_ = .790, 0.433)

a SPSRQ: Sensitivity to Punishment and Sensitivity to Reward Questionnaire.

### 3. Image analyses

#### 3.1 Regional GM differences between normal weight and excess weight groups

**Figure 1:**
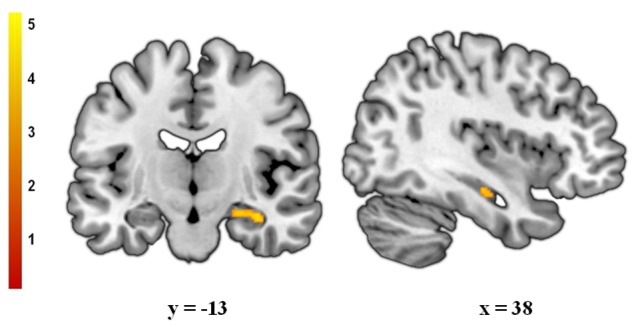
Clusters of significant gray matter volume increase in excess weight compared with normal weight subjects. Peak coordinates were located in the right hippocampus (x, y, *z*_ 38, −13, −18; t = 4.21; pFWE-SVC<0.05). Results are overlaid on coronal and sagittal sections of a normalized brain, and the numbers correspond to the ‘y’ and ‘x’ coordinates in MNI space. Color bar represents t value. For demonstration purposes the images are displayed at p<0.001 (uncorrected, k>50).

ROI analyses reported a significant volume increase in the right hippocampus of excess weight participants in comparison to normal weight subjects (Figure 1). Regarding the whole-brain analyses, there were no significant between-group differences at pFWE<0.05. Nevertheless, at a more lenient significance threshold of p<0.001 (uncorrected, k>250 voxels), we found a significant volume increase in the left precentral region of normal weight subjects (see Figure S1). In addition, in order to further investigate the relationship between BMI and regional GM volumes, we also correlated BMI values against voxel-wise GM volumes, finding no results at a corrected statistical threshold beyond those observed in the qualitative comparisons.

#### 3.2 Correlation analyses with personality and neuropsychological scores

**Figure 2:**
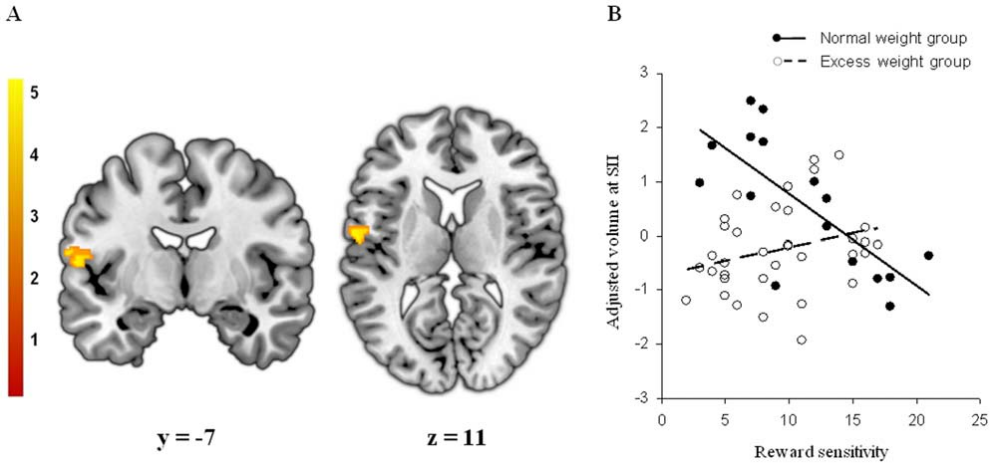
Between-group interaction between regional gray matter volume and reward sensitivity. A. Voxel-wise correlations between regional gray matter volume and reward sensitivity score specifically observed in normal weight subjects. Peak coordinate was located in the left secondary somatosensory cortex (SII, Brodmann area 43) (x, y, z = −60, −7, 11; t = 4.51; pFWE-SVC<0.05). Results are overlaid on coronal (left) and axial (right) sections of a normalized brain, and the numbers correspond to the ‘y’ and ‘z’ coordinates in MNI space, respectively. Color bar represents t value. For demonstration purposes the images are displayed at p<0.001 (uncorrected, k>100). B. Plot of the correlation between gray matter volume at the peak coordinate and the reward sensitivity score. Normal weight group (filled circles, solid line) showed a significant correlation between these two measures (r = −0.750; p<0.005), while in the excess weight group the correlation was not significant (r = 0.284; p>0.05).

**Figure 3:**
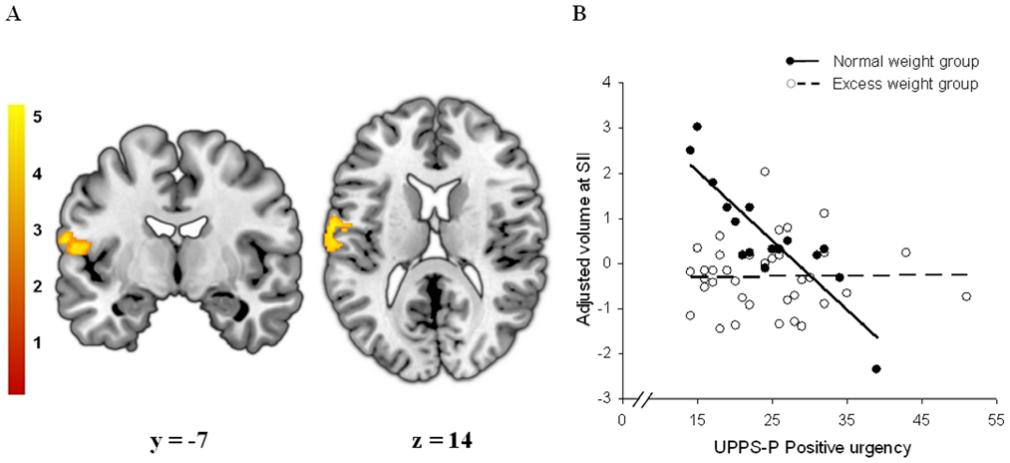
Between-group interaction between regional gray matter volume and positive urgency. A. Voxel-wise correlations between regional gray matter volume and positive urgency (UPPS-P) score specifically observed in normal weight subjects. Peak coordinate was located in the left secondary somatosensory cortex (SII, Brodmann area 43) (x, y, z = −63, −7, 15; t = 4.89; pFWE-SVC<0.05). Results are overlaid on coronal (left) and axial (right) sections of a normalized brain, and the numbers correspond to the ‘y’ and ‘z’ coordinates in MNI space, respectively. Color bar represents t value. For demonstration purposes the images are displayed at p<0.001 (uncorrected, k>100). B. Plot of the correlation between gray matter volume at the peak coordinate and the positive urgency score. Normal weight group (filled circles, solid line) showed a significant correlation between these two measures (r = −0.856; p<0.0005), while in the excess weight group the correlation was not significant (r = 0.058; p>0.05).

**Figure 4:**
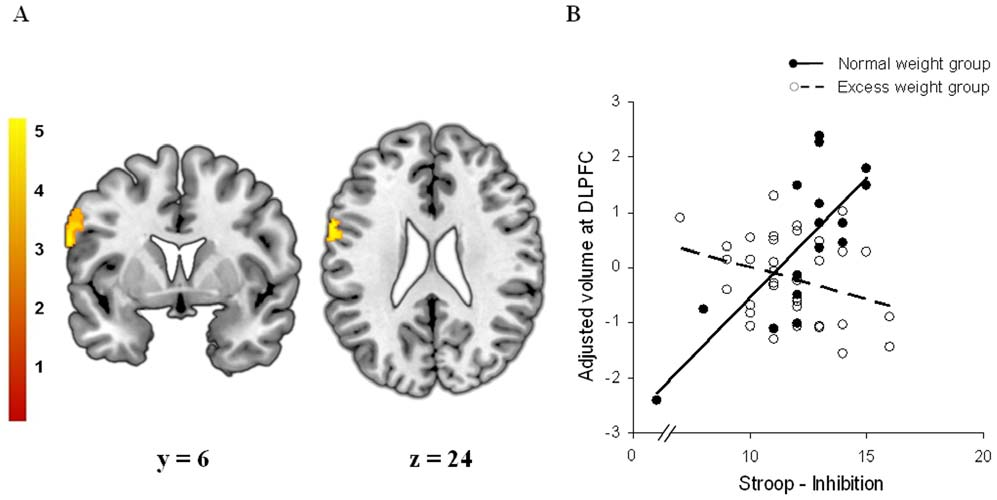
Between-group interaction between regional gray matter volume and response inhibition. A. Voxel-wise correlations between regional gray matter volume and the Stroop response inhibition score specifically observed in normal weight subjects. Peak coordinate was located in the left dorsolateral prefrontal cortex (Brodmann area 9) (x, y, z = −61, 6, 24; t = 5.01; pFWE-SVC<0.05). Results are overlaid on coronal (left) and axial (right) sections of a normalized brain, and the numbers correspond to the ‘y’ and ‘z’ coordinates in MNI space, respectively. Color bar represents t value. For demonstration purposes the images are displayed at p<0.001 (uncorrected, k>100). B. Plot of the correlation between gray matter volume at the peak coordinate and the Stroop response inhibition score. Normal weight group (filled circles, solid line) showed a significant correlation between these two measures (r = 0.769; p<0.005), while in the excess weight group the correlation was not significant (r = −0.327; p>0.05).

Regarding ROI analyses, we found significant correlations between regional GM volumes and the scores of the behavioral tests only in normal weight participants. On the one hand, reward sensitivity and UPPS-P positive urgency scores were negatively associated with the GM volume of the left secondary somatosensory cortex (SII) in control subjects (Table 3 - Figures 2 and 3), whereas these correlations were not observed in the excess weight group. No further correlations were observed with the other personality dimensions. On the other hand, we observed a significant positive correlation between the inhibition score derived from the Stroop test and the volume of the left dorsolateral prefrontal cortex (Table 3 and Figure 4). Again, this correlation was not observed in excess weight participants. No further results were observed in whole-brain analyses.

**Table 3:** Correlations of SPSRQ, impulsivity and inhibitory control scores with brain anatomy in normal weight subjects.

Anatomical region	K	T	pFWE-SVC<0.05	x	y	z
SPSRQ – Reward sensitivity						
*Negative Correlation*						
SII L^a^	267	4.51	0.028	−60	−7	11
UPPS-P – Positive urgency						
*Negative correlation*						
SII L	260	4.89	0.010	−63	−7	15
Stroop – Inhibition						
*Positive correlation*						
DLPFC L^b^	498	5.01	0.006	−61	6	24

a SII L, left secondary somatosensory cortex;

b DLPFC L, left dorsolateral prefrontal cortex. Significant peaks are given in MNI coordinates. The corresponding anatomical names were obtained using the aal toolbox for SPM8.

## Discussion

In this study we aimed to examine voxel-wise differences in regional GM volume between excess weight and normal weight adolescents, and to explore differences in the way reward and punishment sensitivity, impulsivity and inhibitory control related to regional GM volumes in both groups. In partial agreement with initial hypotheses, we found that adolescents with excess weight (the combined group of overweight and obese participants) have structural abnormalities in one predefined ROI, the right hippocampus. Specifically, the excess weight adolescents had increased right hippocampal GM regional volumes compared to lean controls. Furthermore, reward sensitivity and positive urgency scores negatively correlated with left SII regional volumes in lean controls but not in excess weight adolescents. Similarly, Stroop performance scores positively correlated with left dorsolateral prefrontal cortex regional volumes in controls but not excess weight adolescents. In contrast with initial assumptions, we did not find significant alterations in the striatum or the orbitofrontal cortex, or different associations between these regions and personality and cognitive measures.

The finding of an increased right hippocampal volume in excess weight adolescents is in fitting with the role of this region in the processing of motivational signals associated with appetite [26]. For example, functional imaging studies have shown that right hippocampal activation is significantly associated with food cues-induced insulin release in obese adolescents [27] and with direct gastric stimulation in obese adults [13]. Furthermore, the gastric stimulation-induced increases of hippocampal activity were associated with scores of emotional eating and lack of control [13], supporting the role of this region in the incentive motivation and cognitive control of eating behavior in obesity.

Correlation analyses showed that the regional volume of SII was associated with reward sensitivity and positive urgency in lean controls but not in excess weight adolescents. Within SII, the specific region of correlation with reward sensitivity and positive urgency was the subcentral gyrus, or Brodmann area 43, also known as area OP4 [28]. This area occupies the most lateral aspect of SII, adjacent to the representation of the oral cavity within the primary somatosensory cortex, and thus it is mainly involved in the processing of somatosensory information, including the sensory input relevant for gustatory awareness [29], [30]. Interestingly, somatosensory processing regions have been associated with reward sensitivity in healthy individuals with high scores in this personality trait [31]. Moreover, somatosensory regions consistently show increased activations towards food cues in both adolescents at risk of developing obesity [10] and in obese adolescents [10]. The fact that the negative associations of personality measures with SII volume were only observed within healthy controls would suggest that in excess weight subjects the normal function of somatosensory regions in relation to reward sensitivity and impulsivity is missed or hijacked by disease-specific mechanisms. The latter notion would be similar to what is found in addiction, in which drug craving rewires the function of stimulus-valuation and response control brain regions [32], putatively modifying the link between trait impulsivity and brain structure [33]. In this case, the function of SII may be rewired by the persistent activation of somatosensory regions during anticipation or encoding of sensory and hedonic aspects of palatable food, as shown by fMRI studies [10], [11], [34].

Unlike previous studies [8], [9] we did not find significant structural abnormalities in the prefrontal cortex of excess weight adolescents. However, we found a positive association between cognitive inhibitory control (Stroop performance) and a cluster located in the left dorsolateral prefrontal cortex of normal weight subjects. This region has been shown to mediate the link between aerobic fitness and response inhibition in ageing adults, suggesting a link between physical fitness, production of neurotrophic agents (including insulin-like growth factor-1) and protection of higher-order executive skills [35]. Such region may play a similar role in the developing adolescent brain, and thus in terms of individual differences in response inhibition in normal weight adolescents, which is once again absent in the excess weight group. In agreement with such a notion, over-activity of this region during response inhibition has previously been observed in adolescents compared to healthy adult groups [36]. More research is needed to understand why this link is altered in excess weight adolescents, but the impact of adiposity on vascular health and insulin production may particularly impact frontal brain regions and executive functions [37].

The potential limitations of our study include the decision to merge the overweight and obese subgroups, the lack of significant behavioral performance differences, and the lack of significant volumetric differences in a priori regions of interest such as the orbitofrontal cortex and the striatum. The first decision was based on the observation that comparisons between obese and overweight subgroups failed to yield any significant findings. In addition, the study of dimensional measures of adiposity (BMI) did not either add significant results beyond the categorical diagnosis comparison (normal vs. non normal BMIs). Therefore, we consider that these findings actually reflect that the association between BMI and brain anatomy is better captured by a qualitative analysis comparing participants with vs. without clinical problems related to excess weight. With regard to the lack of behavioral differences and of GM differences in the prefrontal cortex and the striatum, we acknowledge that these negative results are somehow opposed to previous findings, and may reflect the fact that our sample was composed of less severe individuals than those of previous studies including higher BMIs and individuals with other comorbidities [9], [38]. In addition, it might be also argued that the unequal number of voxels included in the different ROIs assessed might have favored the detection of significant differences in smaller regions, such as the medial temporal lobe (in opposition to orbitofrontal or dorsolateral prefrontal cortices, for instance). In any case, we also performed a whole-brain analysis, and, even at an uncorrected significances threshold, we only observed a volume decrease in the left precentral region of excess weight participants, but no findings were observed in the prefrontal cortex or the striatum.

In summary, here we report that, in comparison to lean controls, adolescents with excess weight (including participants meeting criteria for overweight and obesity) have increased right hippocampal volume, a brain region related to emotional and motivational aspects of food intake. Somewhat unexpectedly, personality and cognitive measures were mainly correlated with the volume of the second somatosensory region, although significant findings were also observed in the dorsolateral prefrontal cortex in relation to measures of inhibitory control. In any case, the lack of significant differences in the behavioral measures and the fact that correlation analyses grasped some of the potential correlates of adolescent obesity in the prefrontal cortex supports our initial assumption that the assessment of the correlations between neuroimaging and behavioural data is more sensitive than any of these two approaches on its own.

## Supporting Information

Figure S1 **Clusters of significant gray matter volume increase in normal weight compared with excess weight subjects.** Peak coordinates were located in the left precentral region (Brodmann area 6) (x, y, *z*_ −40, −13, 63; t = 4.65; p<0.001 (uncorrected, k>250). Results are overlaid on coronal and sagittal sections of a normalized brain, and the numbers correspond to the ‘y’ and ‘x’ coordinates in MNI space. Color bar represents t value. Voxels with p<0.001 (uncorrected, k>250) are displayed.
(TIF)
