# Supplementary figures and images for: Brain Structural Correlates of Reward Sensitivity and Impulsivity in Adolescents with Normal and Excess Weight
Source: PLoS One. 2012 Nov 21;7(11):e49185. doi: 10.1371/journal.pone.0049185 (PMC3504042; doi:10.1371/journal.pone.0049185)

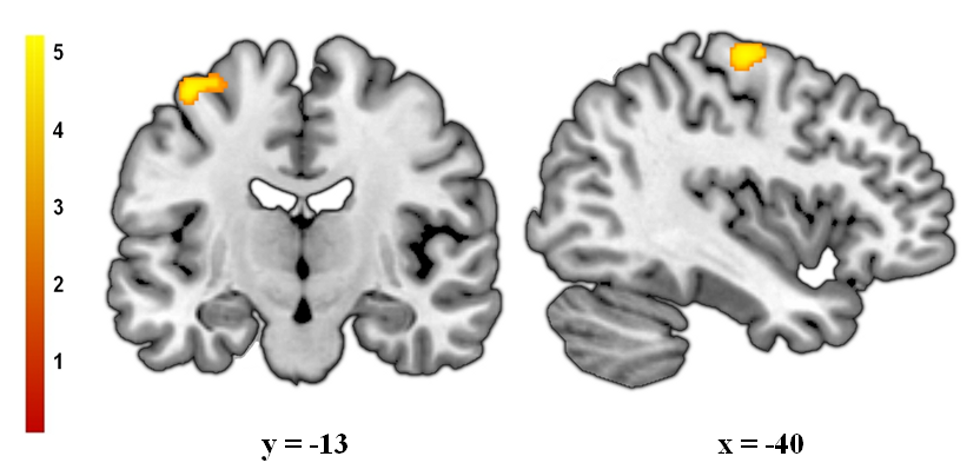

Supplement: Figure S1 — Clusters of significant gray matter volume increase in normal weight compared with excess weight subjects. Peak coordinates were located in the left precentral region (Brodmann area 6) (x, y, z_ −40, −13, 63; t = 4.65; p<0.001 (uncorrected, k>250). Results are overlaid on coronal and sagittal sections of a normalized brain, and the numbers correspond to the ‘y’ and ‘x’ coordinates in MNI space. Color bar represents t value. Voxels with p<0.001 (uncorrected, k>250) are displayed. (TIF) [file pone.0049185.s001.tif]
